# The 4E theory-based performance benchmarking of aged care service provision in community care facilities: a case study of Nanjing, China

**DOI:** 10.3389/fpubh.2025.1467745

**Published:** 2025-02-04

**Authors:** Wei Huang, Ying Hu, Lingzhi Li, Chendi Wang, Luyao Liu

**Affiliations:** ^1^Research Center of Smart City, Nanjing Tech University, Nanjing, China; ^2^School of Civil Engineering, Sanjiang University, Nanjing, China

**Keywords:** community-care, care service provision, performance benchmarking, DEA, social performance, economic performance

## Abstract

The global aging population is growing rapidly, and due to its large population, China is expected to become a ‘super-aged society’ within the next decade. Therefore, ensuring that older people can ‘age in place’ has become a major national priority. Community-care facilities (CCFs) play a key role in this process, but their service performance often faces challenges, with high input costs and limited output benefits. This paper aims to evaluate and improve the social and economic performance of CCFs by proposing a comprehensive framework based on the 4E (Economy, Efficiency, Effectiveness, Equity) theory. Specifically, based on the 4E theory, a literature review and correlation analysis are conducted to select performance indicators. The input–output relationships are then established according to the four dimensions of the 4E theory. These indicators and input–output relationships form the foundation for applying the δ-SBM DEA model, which is used to assess the performance of 75 CCFs in Nanjing. The study finds that the economic performance of CCFs generally lags behind their social performance, with many facilities showing inefficiency in economic indicators, characterized by high operational costs and limited profitability. Projection analysis reveals major issues in low-performing CCFs, including uneven regional distribution of facility performance, poor profitability, a shortage of skilled older people care professionals, and insufficient older people’s participation. Based on these findings, the paper provides specific policy recommendations for government authorities, industry associations, and CCFs, focusing on improving cost-effectiveness, increasing the number of professionals, and enhancing older people’s participation.

## 1 Introduction

The world’s aged population is growing dramatically faster over the past decade (1). Among the many countries experiencing rapid aging, China stands out, with the proportion of people aged 60 and above increasing to 21.10% by the end of 2023 (2). This trend indicates that China will swiftly move toward a “super-aged society” in the next decade (3). Compared to countries like Japan and Germany (4), China has the largest population of older people in the world, which puts immense pressure on its social, economic, and healthcare systems. This demographic challenge makes finding effective solutions to address aging in China even more urgent (5). Therefore, providing care services for older people has become a national priority in China. Given their preference for “aging in place,” the Chinese government has made significant investments in developing community-care facilities (CCFs) to provide services such as healthcare, daytime care, and spiritual support, enabling older people to continue living at home (6, 7). As of 2023, the total number of CCFs in China amounted to 400,000 (8, 9). Despite government support in the form of tax incentives and financial aid, many CCFs still face challenges in sustainable operation. High-performance centers typically achieve higher outputs with lower inputs. In contrast, low-performance centers tend to have higher inputs but relatively average outputs. The imbalance between inputs and outputs is the primary reason for their low performance (10, 11). To address these issues, it is crucial to assess the performance of CCFs from both social and economic perspectives and identify strategies for improvement (12).

In public service performance evaluation, the 3E framework, introduced by Checkland (13), is widely used. It consists of three dimensions: Economy, Efficiency, and Effectiveness (13). The 3E theoretical framework primarily focuses on economic efficiency, assessing how resources are utilized, how efficiently services are delivered, and whether organizational goals are achieved (13). However, as public services are non-profit, their objectives extend beyond maximizing economic benefits. They also aim to address the diverse needs of recipients and ensure fairness. As a result, the model’s neglect of social equity became evident, especially as the diversity of service recipients increased. To address this gap, Flynn expanded the 3E framework in 1997 by adding a fourth dimension-Equity (14). This addition led to the creation of the 4E framework (14). The 4E framework retains the original dimensions of Economy, Efficiency, and Effectiveness, while emphasizing on the fair distribution of resources and opportunities, especially for vulnerable groups (14). In the 4E framework, Economy and Efficiency focus on economic needs, emphasizing the effective use of resources and maximizing output with minimal input. Meanwhile, Effectiveness and Equity focus on the social outcomes of service delivery, ensuring that services are not only effective but also fair and accessible (14). By integrating all four dimensions, the 4E provides a comprehensive and balanced framework for evaluating CCFs performance, meeting both the need for economic efficiency and the demand for social equity (15, 16).

Many previous studies have focused on either the economic or social performance of CCFs, but they often neglected the integration and interrelationship between the two dimensions (17, 18). For instance, many studies highlight the social benefits of care for older people, such as Elsy and Putri’s research on how technological innovations can reduce dependence on human caregivers. However, their study overlooks the long-term cost-effectiveness (19). Similarly, Qin and Yuan’s study addresses the demand-provision imbalance in community-care services in urban China, emphasizing gaps in basic care services. However, it does not explore how policy adjustments or market mechanisms could enhance both economic efficiency and service delivery sustainability (20). To overcome the limitations of existing research, we apply the 4E framework in evaluating CCFs. In the economic dimension, the focus is on maximizing resource output, minimizing costs, and improving service efficiency. Specifically, it examines how to improve economic efficiency through optimal resource allocation, thereby boosting the long-term sustainability of CCFs with limited funding. In the social dimension, the focus is on service accessibility and availability, ensuring that all social groups, especially the older people, can equitably access necessary care services.

For CCFs practices, prior studies made considerable efforts on measuring benchmarking performance via various multi-attribute decision making (MADM) methods, such as analytic hierarchy process (AHP) (21), analytic network process (ANP) (22), and technique for order preference by similarity to ideal solution (TOPSIS) (23). It’s found that these conventional MADM methods solely assess the outcome performance of CCFs services, while they fail to evaluate the effectiveness of input resources. When evaluating performance, it is crucial not only to focus on the final output but also to consider how well input resources are utilized, as CCFs in China are public welfare organizations aiming to maximize resources with limited input. Therefore, a more comprehensive evaluation from an input–output perspective is necessary (24, 25). Data Envelopment Analysis (DEA) is an ideal tool for this purpose because it is a data-driven, non-parametric method that evaluates the efficiency of CCFs with multiple inputs and outputs (26). In addition, traditional performance evaluation methods, such as the CCR, BCC, and SBM models (26–28), have limitations in evaluating complex systems like CCFs, particularly when input–output values are zero or negative. To address this, this paper uses the δ-SBM model, an advanced DEA technique that can effectively handle such data (29).

Based on the aforementioned studies, the objectives of the current study can be summarized as follows:

- This paper applies the 4E theory (Economy, Efficiency, Effectiveness, and Equity) to balance the economic and social dimensions of care services for older people from an input–output perspective.
- This paper aims to assess the performance of 75 CCFs in Nanjing using the δ-SBM model and DEA method, by analyzing the selected input–output indicators.

By integrating the 4E theory with the δ-SBM DEA model, this paper provides a comprehensive evaluation of both the social and economic performance of CCFs, using 75 CCFs in Nanjing as a case study. It also provides specific policy recommendations for government authorities, industry associations, and CCFs.

## 2 Methodology

**Figure 1:**
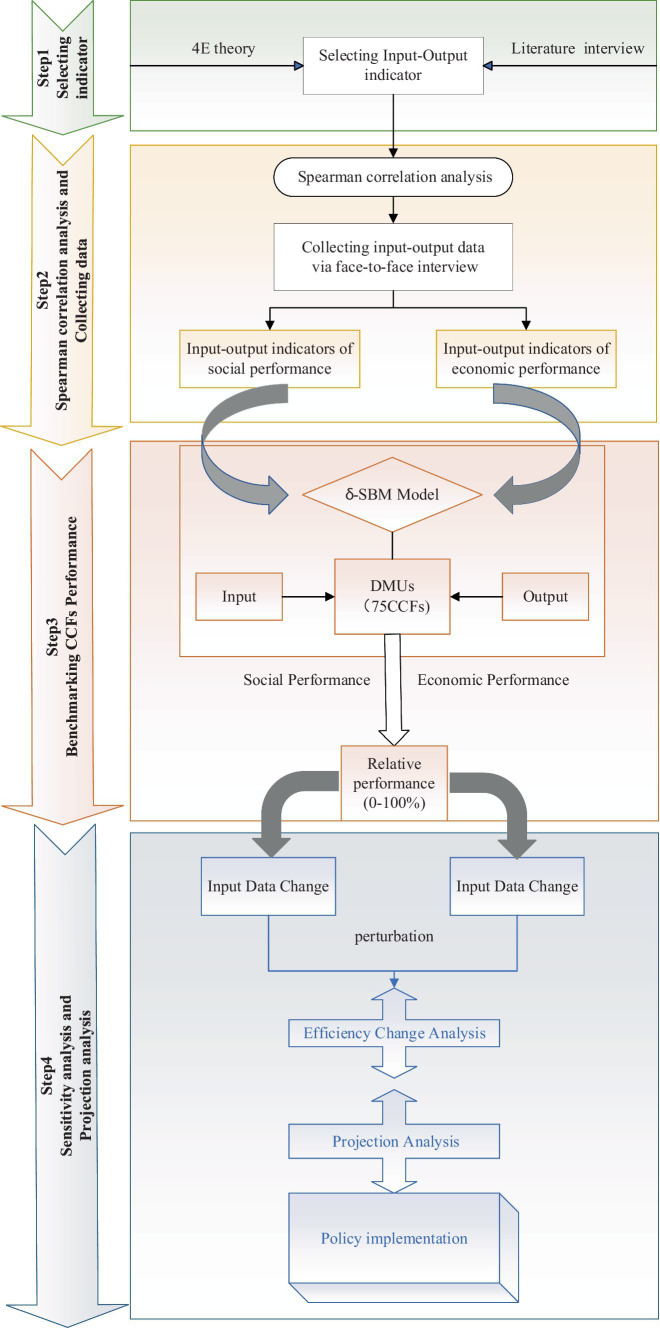
Research process.

The whole procedure of methodology is described in the following four steps (Figure 1).

**Step 1**: Selecting indicator. Based on the 4E theory, this paper identifies potential input–output indicators to represent the economic and social performance of CCFs through a systematic literature review.

**Step 2**: Conducting spearman correlation analysis and collecting data. First, use the Spearman correlation test, logically filter out indicators that are unrelated to operational performance. Then, collect input–output data for each surveyed CCF through face-to-face interviews with facility owners, managers, and nursing service managers. Finally, construct input–output indicators for economic and social performance.

**Step 3**: Benchmarking CCFs performance. After collecting input–output data and confirming the final input–output variables, we used the δ-SBM model to measure the relative performance of CCFs.

**Step 4**: Sensitivity analysis and Projection analysis. To avoid the limitations of DEA, the efficiency changes in the social and economic performance of each CCF are assessed by perturbing the inputs and outputs, which enhances confidence in the model results. Based on the slack variables obtained from the DEA evaluation, the causes of inadequate operational performance in urban CCFs are diagnosed. Projection analysis is conducted for 75 DMUs with lower performance values, and corresponding recommendations are made for government authorities, industry associations, and CCFs.

### 2.1 Model selection

DEA is a nonparametric efficiency evaluation method for assessing the relative efficiency between one or more inputs and outputs (26). The most traditional DEA models are the CCR model (26) and the BCC model. Over the years, DEA models have evolved into various ones such as ADD model (28), SBM model (30), etc. However, the above DEA model may not be able to fully distinguish the performance of individual decision making units (DMUs), which may result in a situation where the performance of multiple DMUs is 1 and cannot be compared, and it is difficult to distinguish the performance of individual DMUs when there are fewer decision making units or more input–output indicators (26–28, 30, 31). To overcome the weaknesses of the traditional DEA models mentioned above, Khezrimotlagh and others proposed a robust δ-SBM model with degrees of freedom, which introduces degrees of freedom and δ-uncertainty to enhance flexibility and improve the discrimination of DMU performance. Unlike traditional DEA models, the δ-SBM model better handles uncertainty and weight allocation, offering a more effective tool for efficiency evaluation. This model has been validated in several studies.

In this paper, the δ-SBM model is applied to evaluate the social and economic performance of CCFs. Given n DMUs (DMU*_i_*, i=1,2,⋯,n ) of m nonnegative inputs ( ${\tilde{x}}_{ij},j=1,2,\cdots ,m$ ), p nonnegative outputs ( ${\tilde{y}}_{ik},k=1,2,\cdots ,p$ ). The δ-SBM model employs the equations and parameters as follows: including Equations 1-1–1-3:


(1-1) $$\max\;\overset{m}{\underset{j=1}{\sum }}w_{lj}^{-}{\tilde{s}}_{lj}^{-}+\overset{p}{\underset{k=1}{\sum }}w_{lk}^{+}{\tilde{s}}_{lk}^{+}$$

subjectto.

(1-2) $$\mathrm{Targets}:\{\begin{array}{c} {\tilde{x}}_{lj}^{*}={\tilde{x}}_{lj}-{\tilde{s}}_{lj}^{-*}+\delta_{j}^{-}\;\\ {\tilde{y}}_{lk}^{*}={\tilde{y}}_{lk}+{\tilde{s}}_{lk}^{+*}-\delta_{k}^{+}\; \end{array}$$

(1-3) $$\mathrm{Score}:{\tilde{\tau }}_{\varepsilon }^{*l}=\frac{\sum_{k=1}^{p}w_{k}^{+}{\tilde{y}}_{lk}/\sum_{j=1}^{m}w_{j}^{-}{\tilde{x}}_{lj}}{\sum_{k=1}^{p}w_{k}^{+}{\tilde{y}}_{lk}^{*}/\sum_{j=1}^{m}w_{j}^{-}{\tilde{x}}_{lj}^{*}}$$

Parameters:

i=1,2,⋯,75 ; DMUs are 75 CCFs.

$w_{ij}^{-}$ , $w_{ik}^{+}$ : 1;

${\tilde{x}}_{lj}^{*},{\tilde{y}}_{lk}^{*}$ : The calculated Performance values;

δ : 0.001;

$\delta_{j}^{-}$ , $\delta_{k}^{+}:$ $\delta_{j}^{-}=0.001\times \min\left(X\right);\;\delta_{k}^{+}=0.001\times \min\left(Y\right)$ ;

min (X) represents the smallest input value among the input data of the 75 CCFs;

min (Y) represents the smallest output value among the output data of the 75 CCFs.

### 2.2 4E-based input–output selection

This paper applies the 4E theory and, through a literature analysis method, preliminarily identifies the performance indicators for CCFs, thereby constructing the performance evaluation indicator system shown in Table 1.

**Table 1:** Performance evaluation indicators identified based on literature review.

Dimension	Code	Quantitative criteria	References
Economy	Construction Costs (C1)	Financial Amount	(40–42)
	Center’s self-raised Construction Costs (C2)	Financial Amount	
	Construction Costs Subsidized by the Government (C3)	Financial Amount	
	Annual Operating Costs (C4)	Financial Amount	
	Center’s Self-raised Annual Operating Costs (C5)	Financial Amount	
	Annual Operating Subsidies Received from Government (C6)	Financial Amount	
	Profitability (C7)	Financial Statements	
Efficiency	Number of Employees (C8)	Personnel Count	(40, 43)
	Number of Volunteers (C9)	Personnel Count	
	Total Personnel Count (C10)	Personnel Count	
	Annual Number of Services Completed (C11)	Service Person-count Quantity	
	Annual Number of Older People Served (C12)	Personnel Count	
	Community-Care Service Coverage Rate (C13)	Ratio of Older People Served to Total Older People in the Street	
Effectiveness	Number of Service Functions (C14)	Service Function Count	(44, 45)
	Older People Satisfaction with Service Experience (C15)	Grading Evaluation	
	Number of Older People with Improved Quality of Life (C16)	Improvement Count	
	Number of Rehired Laid-off Workers (C17)	Personnel Count	
Equity	Overall institutional Development (C18)	Rating	(46–49)
	Job System (C19)	Rating	
	Security System (C20)	Rating	
	Reward and Punishment System (C21)	Rating	
	Feedback System (C22)	Rating	
	Equity in Service Delivery (C23)	Rating	
	Emergency Response Process Development (C24)	Rating	
	Complaint Status (C25)	Rating	

The four dimensions of the 4E theory are defined as follows:

Economy: This dimension emphasizes ensuring the financial sustainability of CCFs through effective resource management, cost optimization, and maintaining a balance between service quality and expenditure. Economic performance in CCFs involves reducing operational costs while providing high-quality services that meet the needs of older population, particularly in a context where resources may be limited and demand is increasing rapidly (32, 33).
Efficiency: Efficiency in CCFs refers to achieving service objectives with the least amount of resources, minimizing waste, and optimizing the use of available facilities, personnel, and technology. In the context of older people care, it involves streamlining operations, improving service delivery speed, and ensuring that the right services are delivered to the right people at the right time. Efficient resource utilization is crucial, especially in light of financial constraints and growing demand for services (27, 30).
Effectiveness: Effectiveness evaluates whether CCFs achieve their primary goals, particularly the improvement of the quality of life for older people. This includes factors such as health outcomes, user satisfaction, and the ability to meet the diverse needs of older people, such as healthcare, social interaction, and emotional support. In addition, the effectiveness dimension also looks at whether CCFs contribute to the well-being of older residents by enhancing their independence and ability to “age in place” (34, 35).
Equity: The equity dimension ensures that older people care services are accessible to all, particularly vulnerable populations, such as low-income older people. It assesses whether resources are distributed fairly, considering the different needs of various groups within the aging population. Additionally, it evaluates the inclusivity of policies and the extent to which they protect the rights of marginalized older groups (36, 37).

**Figure 2:**
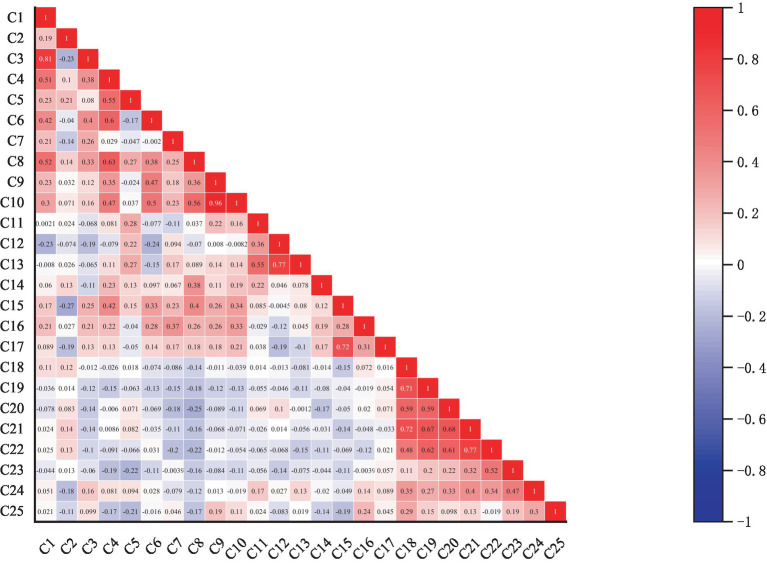
Spearman correlation analysis.

To avoid high correlations between input indicators, which could reduce the DEA model’s ability to distinguish efficient and inefficient units, this paper considers the comprehensiveness of the indicators. Spearman’s correlation analysis is used to select the most representative ones, as shown in Figure 2. The final indicator system for assessing economic and social performance is presented in Tables 2, 3.

**Table 2:** Input–output indicators of economic performance.

	Dimension	Indicators	Code
Input	Economy	Center’s self-raised Annual Construction Costs	E-Input1
		Center’s Self-raised Annual Operating Costs	E-Input2
	Efficiency	Number of Employees in CCFs	E-Input3
	Effectiveness	Number of Service Functions	E-Input4
Output	Economy	Profitability	E-Output1
	Efficiency	Annual Number of Services Completed	E-Output2
	Effectiveness	Older People Satisfaction with Service Experience	E-Output3
	Equity	Equity in Service Delivery	E-Output4
		Complaint Status	E-Output5

**Table 3:** Input–output indicators of social performance.

	Dimension	Indicators	
Input	Economy	Construction Costs Subsidized by the Government	S-input1
		Annual Operating Subsidies Received from Government	S-input2
	Efficiency	Number of Employees in CCFs	S-input3
	Effectiveness	Number of Service Functions	S-input4
Output	Economy	Profitability	S-output1
	Efficiency	Number of Rehired Laid-off Workers	S-output2
		Annual Number of Services Completed	S-output3
		Community Service Coverage Rate	S-output4
	Effectiveness	Older People Satisfaction with Service Experience	S-output5
		Number of Older People with Improved Quality of Life	S-output6
	Equity	Equity in Service Delivery	S-output7
		Complaint Status	S-output8

The economic performance evaluation of CCFs is reflected through the ratio relationships between inputs and outputs, embodying the core goals of economy, efficiency, effectiveness, and equity (38). In the economy dimension, the ratio between inputs such as the center’s self-raised annual construction costs (E-Input1) and operating costs (E-Input2), and outputs like profitability (E-Output1), is used to assess financial sustainability. In the efficiency dimension, the ratio between inputs like the number of employees (E-Input3) and outputs such as annual number of services completed (E-Output2), rehired laid-off workers, and community-care service coverage rate, is used to measure the efficiency of human resource utilization. In the effectiveness dimension, the ratio between the number of service functions (E-Input4) and outputs like older people satisfaction with service experience (E-Output3) and the number of older people with improved quality of life, is used to evaluate goal achievement. In the equity dimension, the ratio between resource inputs and outputs such as service process development (E-Output4) and complaint status (E-Output5) reflects the effectiveness of resource allocation improvements for equitable distribution.

Specifically, the ratio relationships for social performance are as follows: In the economy dimension, we analyze the ratio between government subsidies (S-input1) and profitability (S-output1) to assess the economic efficiency of fund usage; in the efficiency dimension, the ratio between the number of employees (S-input3) and outputs such as service completion, rehired laid-off workers (S-output2), and community-care service coverage rate (S-output4) is used to evaluate human resource efficiency; in the effectiveness dimension, the ratio between the number of service functions (S-input4) and outputs like older people satisfaction with service experience (S-output5) and the number of older people with improved quality of life (S-output6) is used to measure service effectiveness; in the equity dimension, the ratio between resource input and outputs such as service process development (S-output7) and complaint status (S-output8) reflects the improvement of resource distribution fairness. These ratio relationships for both economic and social performance provide a clear evaluation framework for quantifying performance and optimizing resource allocation, enabling a more comprehensive understanding and enhancement of the overall performance of CCFs.

### 2.3 Data collection

The selection of sample CCFs was conducted based on the following criteria: (1) the centers must be officially registered and operating as community-care service providers; (2) the centers must offer at least three different types of older people care services, such as day care, healthcare, and recreational activities; and (3) the centers must be located in different administrative districts of Nanjing, ensuring a diverse representation of geographical and socioeconomic backgrounds.

**Figure 3:**
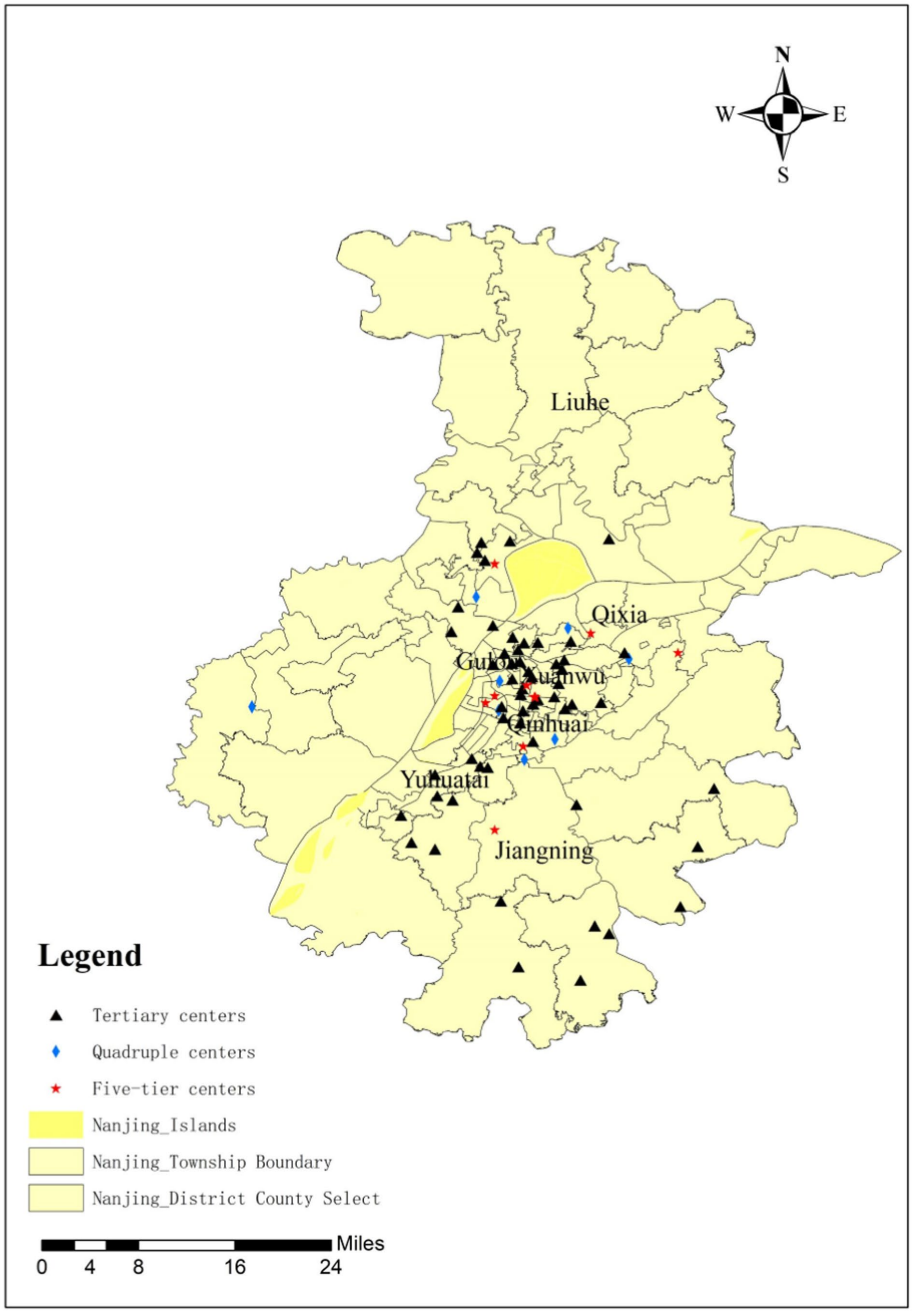
Distribution of 75 CCFs in Nanjing.

Given that Lishui District and Gaochun District are classified as rural areas within Nanjing, this survey concentrated on CCFs in Nanjing’s main urban areas, including Qinhuai District, Pukou District, Qixia District, Jianye District, Yuhuatai District, Jiangning District, and Liuhe District. After making initial telephone contacts, a total of 105 CCFs expressed their willingness to actively participate in the study. Data collection was conducted using a questionnaire that including older people care service provision, service quality, satisfaction, and other relevant factors. Through persistent communication and face-to-face interview, a total of 75 valid responses were ultimately collected, resulting in an overall response rate of 71.4%. This robust response rate reflects a high level of engagement and support from participants, while also enhancing the reliability of the collected data. Using ArcGIS 10.2, a map of 75 CCFs was created, as shown in Figure 3.

### 2.4 Geographically weighted regression analysis

To further ascertain how the research findings vary under different geographical and social-economic contexts, we have added an analysis of the regional economic development levels and performance levels of all 75 CCFs in Nanjing.

Due to the unbalanced development between urban and rural areas in China, there is a lack of data on older people care service centers in rural areas. Therefore, this paper excludes the Lishui and Gaochun districts and focuses on analyzing the data of CCFs in the urban area of Nanjing. Based on a review of relevant literature, combined with our own understanding and considering data availability, this paper uses GDP per capita as an indicator to measure the level of regional economic development. In this paper, only the single dependent variable problem is investigated (39), and thus performs a least squares linear regression analysis in the study: the Equation 1-4 is as follows:


(1-4) $$Y=\beta_{0}+\beta_{1}X+\varepsilon$$

Where *Y* is the dependent variable, *X* is the independent variable, $\beta_{0}$ and $\beta_{1}$ are the parameters of the model, and *ε* is the random error.

Geographically Weighted Regression (GWR) is one of its spatial statistical tools, which can be used to explore the spatial correlation between one or more independent variables and a dependent variable. In this paper, we use the GWR model to further explore the spatial heterogeneity of GDP per capita of each street on the performance level of CCFs. As shown in Equation 1-5, its model expression is as follows:


(1-5) $$y_{i}=\beta_{0}\left(u_{i},v_{i}\right)+\overset{n}{\underset{k=1}{\sum }}\beta_{k}\left(u_{i},v_{i}\right)x_{ik}+\varepsilon_{i}$$

where $y_{i}$ is the dependent variable at location i on a two-dimensional space; $x_{ik}$ is the value of the *k*^th^ independent variable at location i*; n* is the number of independent variables; $\beta_{0}\left(u_{i},v_{i}\right)$ is the intercept parameter at location i, $\beta_{k}\left(u_{i},v_{i}\right)$ is the local regression coefficient for the *k*^th^ independent variable at location i; $\left(u_{i},v_{i}\right)$ are the spatial coordinates of location i; and $\varepsilon_{i}$ is the independent random error at location i.

## 3 Results

### 3.1 Performance results

**Figure 4:**
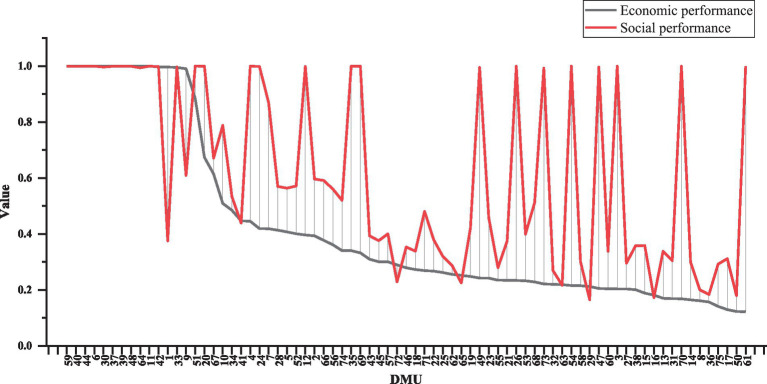
Economic and social performance of 75 CCFs.

The δ-SBM model under the variable return to scale (VRS) condition has been chosen to assess the social and economic performance of the aforementioned 75 decision-making units. According to Figure 4, the 75 CCFs demonstrate distinct trends in economic and social performance. The black line shows a relatively consistent downward trend, indicating that most facilities have similar economic performance, with only slight differences. However, the gap widens toward the lower end, suggesting that some facilities struggle with profitability and resource acquisition, requiring improvements in operational efficiency. In contrast, the red line displays significant fluctuations, reflecting considerable imbalances in social performance. While some facilities excel in service quality, coverage, or user satisfaction, many others suffer from poor service management, insufficient resources, or unmet demand, leading to large disparities within the industry.

Following the statistical analysis, Table 4 presents the efficiency values the 75 CCFs. In general, compared to social performance, economic performance exhibits a higher proportion of 75 CCFs categorized as third-level DEA inefficient, suggesting that current CCFs primarily operate at a level of public welfare with relatively low levels of marketization. However, the differences in social performance more reflect each facility’s ability to serve older populations, the richness of service offerings, and their responsiveness to social needs. Some facilities have greatly enhanced their social impact by innovating welfare programs and volunteer services, boosting social responsibility and equity.

**Table 4:** Performance values of 75 CCFs.

Score range	Number of DMUs (economic)	Number of DMUs (social)	Efficiency value evaluation	
Score = 1	1	4	DEA Efficient	
0.8 ≤ Score < 1	14	24	DEA Invalid	First level
0.4 ≤ Score < 0.8	11	17		Second level
0 ≤ Score < 0.4	49	30		Third level

**Figure 5:**
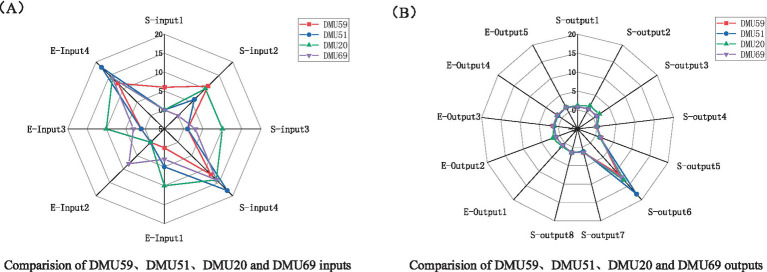
Comparison of DMU59, DMU51, DMU20 and DMU69 inputs and outputs. **(A)** Inputs; **(B)** Outputs.

Among the 75 CCFs surveyed, DMU59 demonstrates effective DEA from an economic performance standpoint, while four facilities-DMU20, DMU39, DMU51, and DMU69-display effective DEA from a social performance perspective. In the subsequent section, we will conduct individual analyses of DMU59, DMU51, DMU20, and DMU69, as illustrated in Figure 5.

DMU59, situated in Xuanwu District, stands as a tertiary CCF distinguished for its diversified enterprise model specializing in home care services. Embracing the ethos of “fostering economic growth through public welfare endeavors and reciprocally supporting public welfare through economic ventures,” this enterprise’s senior care service endeavors are centralized in Nanjing, catering to a population of 210,000 older people. Since its establishment in 2016, despite being small in scale, DMU59 has demonstrated significant efficiency, with one re-employed staff member providing nearly 10,000 services annually. Notably, the facility mainly relies on government subsidies for operation, which cover its operational costs without requiring additional funding. DMU59’s service processes are standardized, labor distribution is clear, and operations are systematic. It performs well in both economic and social performance, closely approaching the effective threshold in the DEA evaluation. Moreover, DMU59’s services extensively cover disadvantaged groups, especially low-income older populations, and have received high social evaluation, demonstrating strong social equity.

DMU51, located in Qinhuai District, is a tertiary CCF showing interesting characteristics. From the first level of DEA inefficiency, its “social performance” is outstanding. However, its economic performance has an effectiveness score of 0.8837, which is slightly below optimal. This facility lacks government construction subsidies. It self-financed construction costs of 30,000 yuan and provides 18 free services to ensure older people receive help. However, due to limited human resources and operating funds, its economic performance is slightly lacking and has not reached optimal levels. Nevertheless, DMU51 demonstrates superior social performance, particularly in its provision of free services, expansion of social welfare coverage, and high levels of recognition and approval from the older population within the community. Its social impact and service equity have been significantly improved.

DMU20 is a five-tier CCF located in Jiangbei New District. It is at the second level of DEA inefficiency, showing good social performance but low economic performance, with an efficiency value of 0.6742. The facility hires four laid-off workers, providing additional employment opportunities for society. Furthermore, its community-care service coverage is extensive, far exceeding the average level. However, from an industry perspective, despite having 10 staff members, it provides fewer than 20,000 services annually, which is inefficient, resulting in a low score for “economic performance.” This facility contributes socially by offering employment opportunities to disadvantaged groups and covering a larger older population than average, demonstrating a high sense of social responsibility.

DMU69, located in Yuhuatai District, provides a comparison of performance indicators. Its “social performance” is considered effective, generating positive benefits; however, its “economic performance” is well below average, with a score of only 0.3324, placing it in the third level of DEA inefficiency. As a private non-enterprise unit, the facility receives no government construction or operational subsidies for social services. Due to limited government investment, its social performance is relatively high. However, the facility’s investors seek more financial support and adjustments to promote a virtuous cycle of operation and enhance services for older people. Despite its insufficient economic performance, the facility plays a positive role in providing care for older people, especially in enhancing social participation and mental health, reflecting its significant social impact.

**Figure 6:**
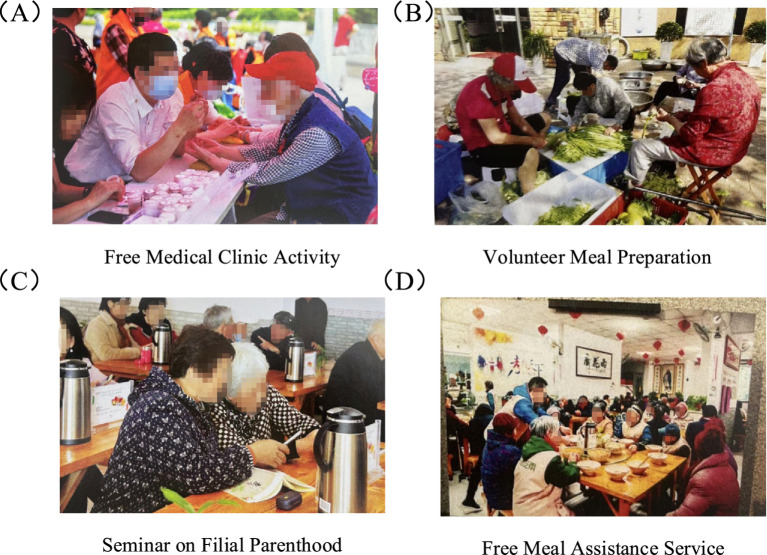
Details of DMU27. **(A)** Free medical clinic activity; **(B)** Volunteer meal preparation; **(C)** Seminar on filial parenthood; **(D)** Free meal assistance service.

DMU27, located in Jiangbei New District, is a quadruple center that started with promoting traditional culture and is also one of the district’s community-care integration innovation pilot projects. It provides 20,000 services annually to older people, which is quite remarkable, but its social and economic performance is poor. Through its public mutual aid restaurant, the facility has been providing free meals to older people for many years, along with cultural enrichment, psychological support, and health education activities. It has also planned various activities, including filial piety lectures and charitable clinics (Figure 6). However, research shows that its 19 activities are provided free of charge, placing a heavy burden on self-financing. Nevertheless, DMU27 continues to provide substantial free social services, demonstrating its strong social responsibility and public welfare value.

In essence, high-performing CCF distinguish themselves by achieving notable outputs relative to their inputs. This might entail maintaining a lean workforce and offering a limited number of service functions while catering to a substantial volume of annual service visits. Alternatively, it could involve generating outputs that surpass inputs, such as facilitating reintegration into the labor force for a higher number of individuals or providing a greater number of free functions and service completions annually. As for low-performing facilities, whether from the perspective of the senior care economic or from the perspective of the social, the higher inputs and more general outputs are the main reasons for the lower performance of these facilities. Consequently, the challenge confronting CCFs lies in effectively translating inputs into commensurate outputs. This demands strategic reevaluation and optimization of resource allocation to enhance overall operational efficiency and efficacy.

### 3.2 Sensitivity analysis

**Figure 7:**
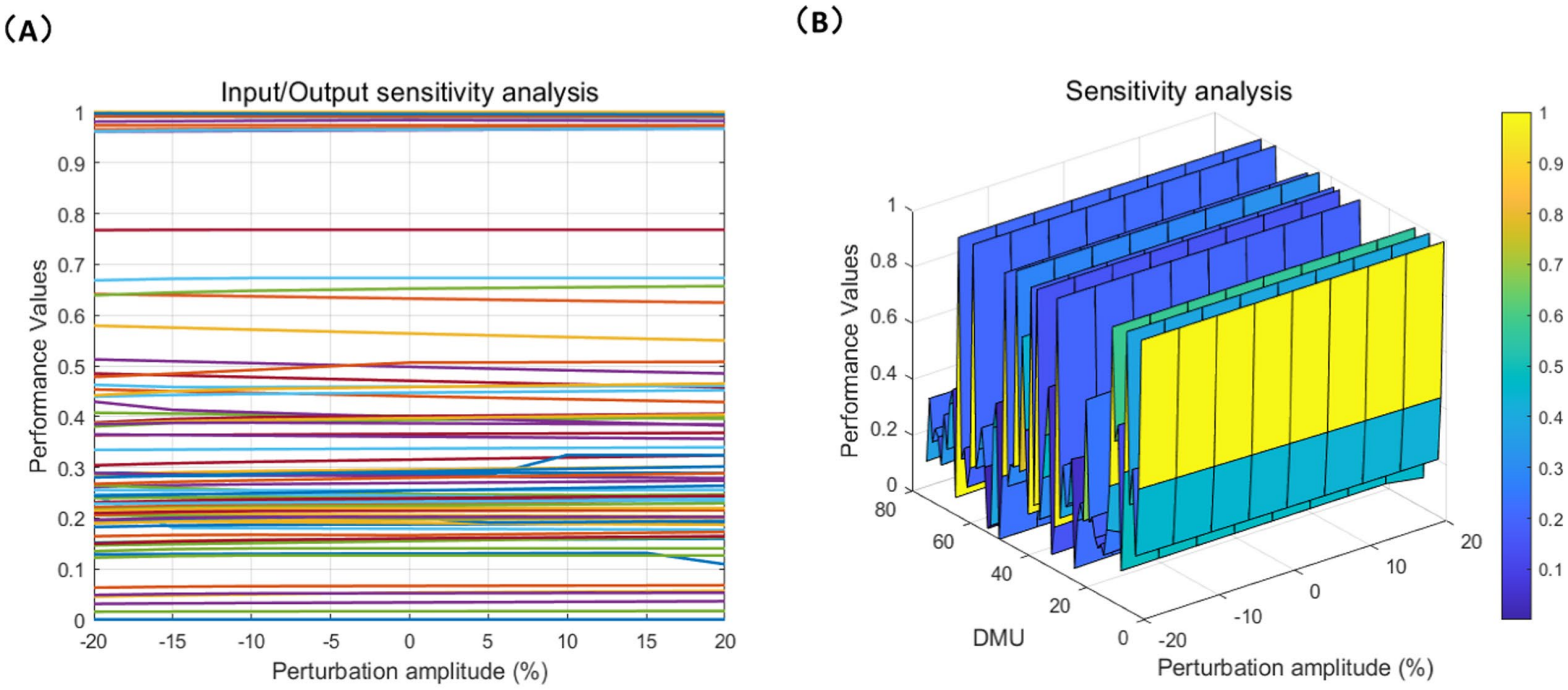
Economic indicators sensitivity analysis. **(A)** Line chart; **(B)** Mesh chart.

**Figure 8:**
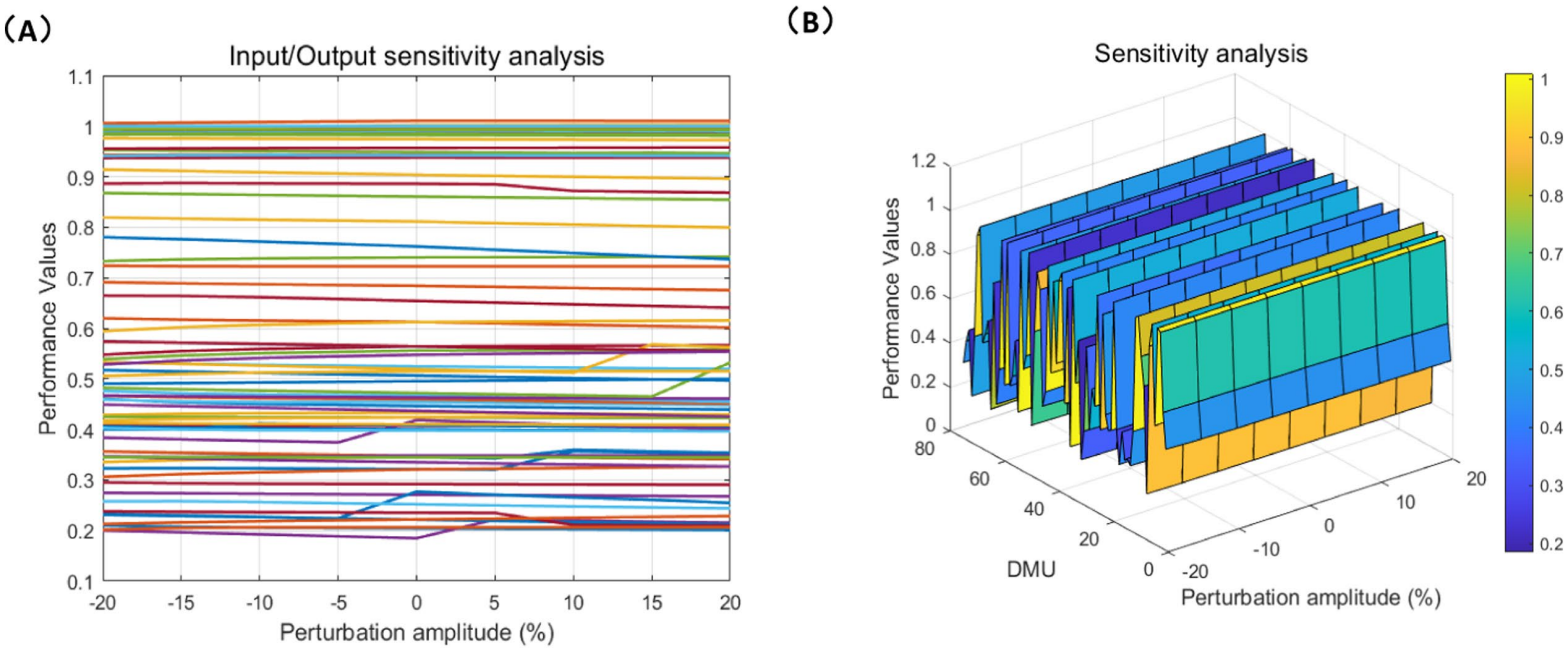
Social indicators sensitivity analysis. **(A)** Line chart; **(B)** Mesh chart.

The purpose of sensitivity analysis is to evaluate the efficiency changes of each DMU by perturbing the inputs and outputs. We attempt to evaluate the efficiency changes of each DMU under different conditions, which helps us identify, which inputs (such as Center’s self-raised Annual Construction Costs, etc.) and outputs (such as service completion, profitability, etc.) have a greater impact on the overall performance of CCFs. Figures 7, 8 demonstrate the sensitivity of both social and economic performance to the perturbation magnitude through line charts and mesh charts.

The sensitivity differences between economic and social performance are further validated by the results of the DEA model, which reveal disparities in these facilities’ resource allocation, management levels, and service quality. Figures 7, 8 show that the efficiency changes of the 75 DMUs under input and output perturbations are minimal, demonstrating strong robustness, indicating that their efficiency is stable in the face of external disturbances. High-performance facilities typically have optimized resource allocation and management capabilities, allowing them to maintain stable performance when faced with external changes. In contrast, low-performance facilities, due to resource shortages or poor management, are more susceptible to external disruptions. These sensitivity differences confirm the results of the DEA model, which show that economic and social performance are influenced by multiple factors, and the differences in performance reflect the varying strengths and weaknesses of facilities in resource allocation, management, and care service provision.

## 4 Discussion and recommendations

Based upon the empirical findings mentioned previously, this paper further diagnoses the reasons for the insufficient operational performance of urban CCFs, and carries out a benchmarking analysis of decision-making units with low performance values, as shown in Supplementary Tables S1, S2. Finally, based on the results of the analyses, corresponding recommendations are made to relevant government departments and industry associations, and CCFs, respectively.

### 4.1 Projection analysis of the economic performance of CCFs

In terms of input indicators, 22 facilities show personnel redundancy, with DMU62 having the highest redundancy rate at 88.43%. Notably, DMU62 has 18 employees, one of the largest workforces among its peers. Despite the large number of staff, inefficiencies arise due to unclear role definitions. As one facility manager mentioned, “We have a large team, but the roles aren’t always clearly defined, leading to overlapping responsibilities. It’s hard to match the right people with the right tasks, which means we are paying for staff we do not fully utilize.” Therefore, improving staff composition and clarifying roles are seen as essential first steps in improving facility performance. Additionally, 53 facilities show redundancy in their self-financed building expenses, with DMU24 having a 100% redundancy rate. One facility manager explained, “We’re located in a remote area, and the facility is smaller than others in the city. While the government funding covers some costs, we still need to invest in infrastructure. But because of our location, we cannot generate enough income to cover the additional expenses. It’s a tough situation.” This aligns with the quantitative findings, indicating that socio-environmental factors, such as facility size and location, contribute to financial inefficiencies. Regarding government operating subsidies, 26 out of 37 facilities show redundancy rates exceeding 80% or even 100%. The manager of DMU27 shared, “The subsidies are helpful, but they aren’t enough to cover the growing demand for services. We often have to look for other funding sources to fill the gap. The system is in place, but it needs to be more responsive to our needs.” This highlights that while subsidy systems exist, their allocation and flexibility need to be improved to better meet the operational needs of facilities. In terms of output indicators, many facilities display deficiencies in service quality, particularly in areas such as the number of services completed, older people care satisfaction, service process perfection, and complaint handling. For DMU20, the manager stated, “We’ve had complaints about our service quality, and some clients feel like they are not getting the attention they deserve. We need to improve our processes and staff training to meet expectations.” This aligns with the quantitative analysis, showing that DMU20 has significant deficiencies in these key performance indicators. Similarly, DMU27 has deficiencies in areas such as annual financial status, older people service satisfaction, and complaint handling. Despite its annual financial status exceeding projections at 965.20%, this has not translated into improved service performance. As the manager of DMU27 noted, “Our financial situation looks better on paper, but it has not translated into better service quality or sustainability. We need to focus on managing our funds better and engage more with our community to attract more clients.” This underscores the need to improve financial efficiency and service utilization.

Overall, while the quantitative analysis highlights issues such as personnel redundancy and misallocation of financial resources, the qualitative insights from interviews provide deeper context and explain the underlying causes. Financial performance often fails to translate into better service quality, and there are inefficiencies in both staff composition and subsidy utilization. Therefore, strategies to improve facility performance should include optimizing staff structure, improving financial management, enhancing service quality and customer satisfaction, and strengthening marketing efforts to increase service utilization and attractiveness. These combined measures can effectively improve the operational efficiency and economic effectiveness of CCFs.

### 4.2 Projection analysis of the social performance of CCFs

Regarding input metrics, 36 facilities show redundancy in government-subsidized expenses annually, with DMU32, DMU36, and DMU63 exhibiting 100% redundancy. Additionally, 41 facilities display redundancy in government-funded operating subsidies annually, with DMU15 having the greatest redundancy rate at 78.70%. One facility manager shared, “The subsidies help, but they are not enough. We still have to look for other funding sources to cover costs, and sometimes the allocation is not responsive enough to our actual needs.” This highlights the inefficiencies in the current subsidy system, suggesting that a more tailored and responsive approach is necessary. From an output standpoint, most facilities exhibit significant shortcomings. Notably, DMU50 has deficiencies across all output metrics. Located in Qinhuai District, a region with a substantial aging population, DMU50 faces a particularly challenging situation. Despite being in an area with an aging population of 27.50% and nearly 20,000 older residents, its number of services completed annually is only about 300, equating to a mere 0.17%. As one of the staff members explained, “We are located in a district with many older people, but our service scale is too small. We do not have enough capacity to meet the needs of the aging population here, which directly impacts our performance.” This undersized service scale is a major factor contributing to the facility’s underperformance. To address this, the facility must take proactive steps to increase its visibility and attract older people to its services. “We need to do more marketing, engage with older people more actively, and improve the awareness of our services,” the manager emphasized. Currently, CCF services mainly cover meal and bathing assistance, age-appropriate renovations, and door-to-door services, primarily delivered by community workers, volunteers, and older people service organizations. As one service provider noted, “The older population’s needs are not uniform. Some need medical care, others need companionship or help with daily tasks. It’s crucial to have a variety of services to meet these different demands.” This points to the necessity of a multi-modal strategy to improve the performance of CCFs. Moreover, from a social perspective, the distribution of government subsidies should consider the unique needs and circumstances of each facility to ensure fair and efficient use. “Each facility is different, and the subsidies should be more flexible to match the local needs,” one local government official suggested.

From both economic and social perspectives, it is critical to implement proactive measures to attract older people to use the services of these facilities, thereby increasing service utilization and overall satisfaction. This implies expanding service offerings, maintaining high standards, and prioritizing the improvement of service quality for older people. “We cannot just wait for people to come; we need to go out and engage them, understand their concerns, and show them the benefits of using our services,” another manager emphasized. By focusing on these aspects, the overall effectiveness and performance of the centers can be significantly improved.

### 4.3 Geographically weighted regression results

The results of the regression analysis can be seen in Table 5. *p*-value is 0.069, which is less than 0.1, and the regression coefficient is −0.321, indicating that the level of regional economic development is negatively correlated with the level of CCFs in each street at the 10% significance level. That is, higher income levels among residents may be associated with an increase in welfare resources and access opportunities in the region. Additionally, the expansion of external welfare services may reduce the demand for home care services for older people, potentially hindering the improvement of CCF performance. This can manifest in a reduction in the number of services provided and a narrowing of the range of service offerings. However, these explanations are speculative and not the focus of the present study.

**Table 5:** Results of univariate analysis of external factors.

Variable	Code	Beta	*p*
GDP per capita	x_1_	−0.321	0.069

**Figure 9:**
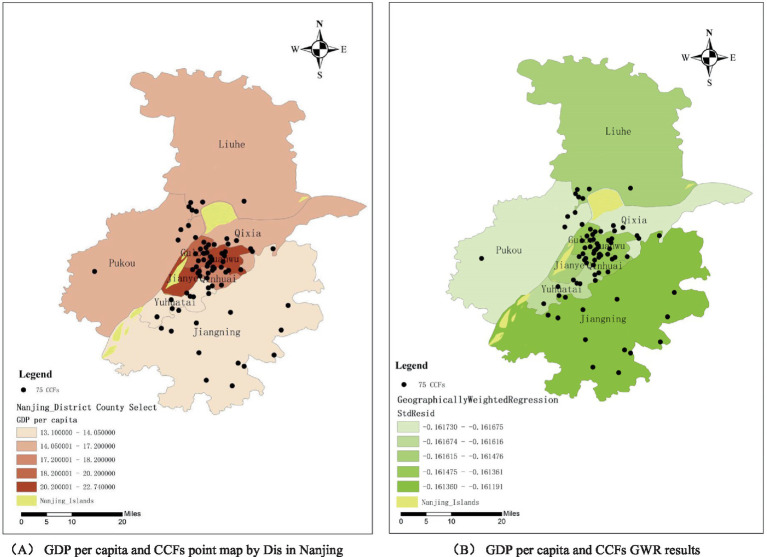
Geographically weighted regression results. **(A)** GDP per capita and CCFs point map by Dis in Nanjing; **(B)** GDP per capita and CCFs GWR results.

Figure 9A shows the distribution of GDP per capita in the main urban areas of Nanjing, overlaid with the distribution of the 75 CCFs points. In regions with higher GDP per capita, the performance level of CCFs tends to be lower. This result confirms the conclusion derived from the aforementioned significance analysis, which suggests that the increase in the level of regional development limits the improvement of CCFs’ performance levels. According to Figure 9B, the regression coefficient interval between GDP per capita and CCFs’ performance levels across different regions is [−0.161730, −0.161191], consistent with the linear regression result where the regression coefficients are all negative. Additionally, the small variation in the regression coefficients indicates that the influence of GDP per capita on CCFs’ performance levels is relatively stable across regions. These results reveal that the spatial heterogeneity for CCFs’ performance is low.

### 4.4 Findings and recommendations

Extracted from the aforementioned discussion, government resources allocated for older people have seen a gradual increase. In addition, even though CCFs perform admirably in social domains, their economic performance is relatively poor. The major findings and implications are described as follows:

1. The performance of facilities is unevenly distributed across regions. This uneven distribution may stem from imbalances in regional policies and development levels, where managers of high-performing CCFs tend to prioritize the development of facilities in certain areas.
2. CCFs have poor profitability. When CCFs face financial difficulties, they often reduce services or service quality, leading to a decline in service levels, which negatively impacts older residents’ satisfaction and experience. Moreover, financial constraints prevent the expansion of services, limiting the number of older people who can benefit from care. Additionally, low profitability may result in salary cuts or delays, affecting staff income and working conditions, thus creating a negative cycle that undermines service quality.
3. There is a shortage of skilled older people care professionals. There is a significant shortage of qualified older people care professionals, and the quality of their work is generally low. This limits the ability of CCFs to improve service quality and negatively impacts the number of older people served annually, hindering economic optimization. For example, DMU20, a facility ranked in the second level of DEA inefficiency in the “older people care industry” perspective, provides fewer than 20,000 services per year with just 10 employees. This is one of the primary reasons for its underperformance. The older people care industry also suffers from a lack of standardization and regulation, with no uniform criteria for evaluating professional competence, leading to significant variation in service quality across facilities.
4. There is insufficient older people’s participation in CCFs. The older people population is not highly motivated to engage with CCFs, resulting in fewer service visits and lower coverage of services in communities, which leads to inefficiencies in resource utilization. This may be attributed to differences in lifestyle and awareness. Many older people are accustomed to family-care and lack knowledge or trust in external CCFs, requiring time and effort to adapt. Additionally, self-perception issues may also contribute. Many older people may feel they are not yet in need of aging-in-place services, and prematurely participating in such services may negatively impact their self-esteem. As a result, low service quality and inadequate coverage may lead to dissatisfaction, causing older people to disengage from these services altogether.

**Figure 10:**
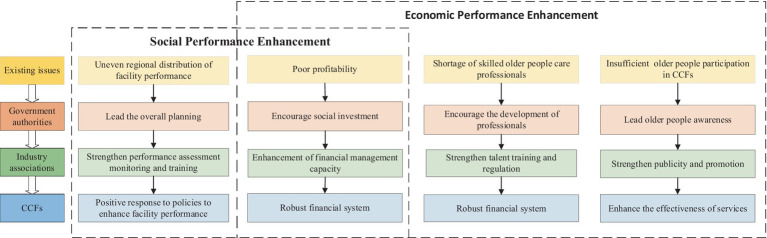
Policy suggestions for improving the social and economic aspects of older people care.

This research suggests the following recommendations based on the analysis of uneven regional distribution of facility performance, poor profitability of CCFs, a shortage of skilled older people care professionals, and insufficient older people’s participation in CCFs (Figure 10).

First, facility development plans should be carefully crafted by government authorities to accommodate changing needs of aging populations and the current configuration of CCFs throughout administrative regions. Through deliberate coordination of the geographic dispersion and magnitude of CCFs, we can guarantee thorough coverage and efficient use of resources. Furthermore, improving the CCF’s incentive and subsidy policy is vital. We enable CCFs to improve their quality and operational effectiveness by strengthening financial support and offering incentives, so that they may better meet the changing demands of the senior population. Proactive policies should also be developed to draw and keep talent in the CCFs. By implementing focused policy, we can encourage professionals to provide community-care and create an atmosphere that supports the development and retention of talent. Moreover, government initiatives should actively engage the public in raising awareness about community-care. By disseminating information, launching awareness campaigns, and formulating policies to promote its importance and benefits, we can foster widespread public participation and acceptance of community-care services.

Second, industry associations should comprehensively enhance the development and performance of CCFs through measures such as market-oriented operation, manpower training and supervision, publicity and promotion, and participation by older people. To begin with, promote the market-oriented operation of CCFs through the introduction of social investment to enhance service quality and efficiency, and encourage cooperation with medical institutions and rehabilitation centers to achieve resource sharing and cost reduction so as to raise the level of service specialization. Moreover, organizing professional skills training courses to improve the professional abilities and quality of service personnel in areas such as older people care and medication management, encouraging joint training between older people care facilities and other institutions, and implementing a system of subsidies and incentives to promote the development of human resources, while at the same time establishing a regulatory and assessment mechanism to ensure the quality of services. Lastly, it conducts regular publicity campaigns to disseminate information and service introductions to attract the active participation of older people; it organizes cultural and recreational activities to improve the diversity and quality of services, and it establishes a feedback mechanism on demand to provide personalized services to ensure that older people receive a satisfactory service experience.

Third, for the core operations of CCFs, it’s imperative to establish a standardized financial management system tailored to the unique circumstances of each facility. This system will maintain a sound profitability, manage fund expenditures carefully, and maximize the efficiency of fund utilization. To enhance the senior population’s leisure time, a wide range of cultural, recreational, and health activities has to be planned. By broadening the range of services provided, these events can be made available to senior citizens and their families who are in need, encouraging greater engagement and excitement from the senior population. Moreover, it is imperative that military personnel receive more intensive operational training. The professionalism and skill level of service personnel should be raised through regular training sessions and knowledge sharing. Through these initiatives, service quality can be enhanced, ensuring the delivery of exemplary care and support to older people population.

## 5 Conclusion

This paper adopts the 4E (Economy, Efficiency, Effectiveness, and Equity) theory to balance the economic and social dimensions of older people care services from an input–output perspective. Based on the δ-SBM model, 75 CCFs in Nanjing are set as DEA assessment DMUS. The collected and screened indicator data are input into the DEA assessment model to obtain the relative performance of the DMUs and analyze the performance of the CCFs through these indicators. To overcome the limitations of the DEA model, this paper combines sensitivity analysis to identify which factors have a greater impact on the results, thereby improving the reliability and accuracy of the decisions. Additionally, to further examine how the research results vary across different geographical or socio-economic contexts, the manuscript includes an analysis of the regional economic development levels and performance levels of the 75 CCFs in Nanjing. Finally, based on the DEA projection analysis, this paper identifies the internal influencing factors of operational performance in urban CCFs, such as redundant input indicators and insufficient output indicators, and summarizes the reasons for the insufficient operational performance of urban CCFs, providing corresponding policy recommendations for government authorities, industry associations, and CCFs.

This paper set up a system of key operational performance indicators for CCFs in the same dimension according to the characteristics of economy and society, and clarify the redundant input indicators and insufficient output indicators affecting the operational performance of urban CCFs, so as to optimize the performance of CCFs in the same dimension. From the input–output perspective, this paper further construct a panoramic process and methodology of “performance target identification- performance indicator selection-performance evaluation-performance optimization” to evaluate the operational performance of urban CCFs.

The current research mainly relies on data collected through manual interviews for performance evaluation, which is inefficient and has a limited sample size. Therefore, future research should leverage big data technology to establish an intelligent older people care service platform, improving the accuracy and operability of performance evaluations through a data-driven approach. The current research focuses mainly on CCFs in urban areas, while the service gap between urban and rural areas remains a significant issue. The rural older people population is large and highly concentrated, making older people care in rural areas a key challenge in addressing aging. Future research should explore the performance evaluation of CCFs in rural areas, focusing on service delivery, resource allocation, and the urban–rural gap. By comparing the performance of CCFs in urban and rural areas, key issues can be identified, and improvement strategies can be proposed to promote balanced development of older people care services.

## Data Availability

The original contributions presented in the study are included in the article/Supplementary material, further inquiries can be directed to the corresponding author.
