# Supplementary material for: The 4E theory-based performance benchmarking of aged care service provision in community care facilities: a case study of Nanjing, China
Source: Front Public Health. 2025 Feb 4;13:1467745. doi: 10.3389/fpubh.2025.1467745 (PMC11834519; doi:10.3389/fpubh.2025.1467745)
Supplement: Supplementary file 1 [file Table_1.docx]

SUPPLEMENTARY APPENDICES

Catalogue

[Supplementary Table S1: Projection Analysis of the Economic Performance (%) 1](#_Toc185605464)

[Supplementary Table S2: Projection Analysis of the Social Performance (%) 5](#_Toc185605465)

Supplementary Table S1: Projection Analysis of the Economic Performance (%)

**Table S1 Projection Analysis of the Economic Performance (%)**

|  | Input redundancy rate | | | | Output shortfall rate | | | | |
| --- | --- | --- | --- | --- | --- | --- | --- | --- | --- |
|  | E-Input1 | E-Input2 | E-Input3 | E-Input4 | E-Output1 | E-Output2 | E-Output3 | E-Output4 | E-Output5 |
| DMU1 | 0 | 0.24 | 0.00 | 0.00 | 0.59 | 0.00 | 0.09 | 0.42 | 0.42 |
| DMU2 | **72.22** | 0 | **45.40** | 0.00 | 67.74 | 0.00 | 169.57 | 8.40 | 27.68 |
| DMU3 | **87.57** | 100.00 | **75.05** | 0.00 | 39.48 | 0.00 | 65.39 | 159.21 | 80.01 |
| DMU4 | **77.42** | 0 | **27.47** | 0.00 | 57.09 | 0.00 | 154.97 | 0.07 | 18.60 |
| DMU5 | **49.95** | 0 | 0.00 | 0.00 | 36.85 | 350.71 | 100.03 | 24.00 | 12.03 |
| DMU6 | 0.00 | 0 | 0.03 | 0.00 | 0.05 | 0.00 | 0.13 | 0.07 | 0.08 |
| DMU7 | **81.67** | 0 | 31.71 | 0.00 | 60.22 | 0.00 | 163.46 | 0.07 | 19.61 |
| DMU8 | **100.00** | 0.00 | **55.38** | 0.00 | 301.69 | 600.01 | 121.38 | 186.63 | 186.63 |
| DMU9 | 0.00 | 0 | 0.19 | 0.00 | 0.00 | 0.00 | 4.07 | 0.00 | 0.71 |
| DMU10 | **63.15** | 0 | 9.03 | 0.00 | 61.05 | 0.00 | 151.42 | 8.40 | 25.51 |
| DMU11 | 0 | 0.03 | 0.00 | 0.00 | 0.03 | 0.00 | 0.29 | 0.11 | 0.11 |
| DMU12 | **98.00** | 0 | **38.90** | 0.00 | 22.74 | 0.00 | 101.55 | 33.40 | 28.57 |
| DMU13 | **99.42** | 100.00 | **52.03** | 0.00 | 215.29 | 0.00 | 123.66 | 127.26 | 126.22 |
| DMU14 | **85.00** | 100.00 | 0.00 | 0.00 | 36.37 | 766.05 | 72.75 | 147.91 | 112.03 |
| DMU15 | **94.79** | 100.00 | 0.00 | 0.00 | 5.80 | 494.12 | 111.13 | 137.58 | 112.66 |
| DMU16 | **53.27** | **100.00** | 0.00 | 0.00 | 0.00 | **959.51** | **95.05** | **85.90** | **60.49** |
| DMU17 | **99.74** | **100.00** | 0.00 | 0.00 | **888.93** | **259.51** | **120.30** | **83.40** | **81.78** |
| DMU18 | **63.53** | 0 | 0.00 | 0.00 | **46.47** | **671.69** | **37.69** | **108.40** | **80.25** |
| DMU19 | **84.98** | **100.00** | 0.00 | 0.00 | **89.49** | **378.73** | **72.75** | 25.07 | 17.82 |
| DMU20 | 8.31 | 0 | 0.00 | 0.00 | 0.00 | **84.21** | **36.45** | **48.08** | **52.60** |
| DMU21 | **87.01** | **100.00** | 0.00 | 0.00 | 1.33 | **425.75** | 11.05 | **113.45** | **84.23** |
| DMU22 | **95.82** | 0 | 0.00 | 0.00 | **49.50** | **241.39** | **333.37** | **58.40** | **91.28** |
| DMU23 | **92.61** | **100.00** | 0.00 | 0.00 | **53.84** | **423.91** | 0.00 | **75.94** | 17.78 |
| DMU24 | **100.00** | 0 | 0.00 | 49.95 | 0.12 | 0.10 | 24.08 | 0.16 | 73.00 |
| DMU25 | 0 | 0 | **33.38** | 0.00 | **633.48** | **90.49** | **300.12** | **33.40** | **33.40** |
| DMU26 | **96.82** | **100.00** | 16.63 | 0.00 | **193.83** | 0.00 | **99.61** | **127.26** | **76.95** |
| DMU27 | 0.00 | 97.41 | 0.00 | 0.00 | **965.20** | 0.00 | **114.28** | **137.41** | **143.26** |
| DMU28 | **49.95** | 0 | 0.00 | 0.00 | **36.85** | **335.51** | **100.03** | 24.00 | 12.03 |
| DMU29 | 0.00 | **92.16** | 0.00 | 0.00 | **912.56** | **55.54** | **185.57** | 76.46 | 84.14 |
| DMU30 | 0 | 0.02 | 0.01 | 0.00 | 0.00 | 0.13 | 0.03 | 0.06 | 0.08 |
| DMU31 | **63.84** | **67.27** | 0.00 | 0.00 | 9.60 | **1327.12** | 0.00 | **71.80** | **83.64** |
| DMU32 | 17.03 | **79.08** | 0.00 | 0.00 | **112.31** | **942.93** | 0.00 | **98.31** | **73.86** |
| DMU33 | 0.05 | 0.05 | 0.02 | 0.00 | 0.00 | 2.63 | 0.00 | 0.02 | 0.02 |
| DMU34 | **99.15** | 0 | **38.92** | 0.00 | 0.00 | 0.00 | 16.13 | **35.72** | 5.51 |
| DMU35 | 0.00 | **100.00** | **47.60** | 0.00 | **46.51** | **344.20** | 5.30 | **31.32** | 0.00 |
| DMU36 | **76.51** | **100.00** | 0.00 | 0.00 | **176.89** | **633.13** | **229.75** | **105.18** | **138.53** |
| DMU37 | 0.03 | 0.04 | 0.03 | 0.00 | 0.00 | 0.19 | 0.00 | 0.06 | 0.05 |
| DMU38 | **72.49** | 0 | 0.00 | 0.00 | **66.67** | **986.14** | **65.23** | **125.07** | **146.00** |
| DMU39 | 0.03 | 0.03 | 0.04 | 0.00 | 0.00 | 0.17 | 0.04 | 0.05 | 0.05 |
| DMU40 | 0 | 0.00 | 0.01 | 0.00 | 0.03 | 0.00 | 0.07 | 0.07 | 0.07 |
| DMU41 | **92.49** | 0 | 0.00 | 0.00 | 9.10 | **170.00** | 23.93 | 25.07 | **46.00** |
| DMU42 | 0.00 | 0.43 | 0.00 | 0.00 | 0.00 | 0.00 | 0.33 | 0.38 | 0.35 |
| DMU43 | 0.00 | **30.22** | 0.00 | 0.00 | **358.66** | 3.56 | **242.27** | **191.20** | **197.01** |
| DMU44 | 0.00 | 0 | 0.00 | 0.00 | 0.02 | 0.00 | 0.06 | 0.07 | 0.08 |
| DMU45 | **45.55** | 0 | **40.72** | 0.00 | 0.00 | **632.62** | 0.00 | **33.59** | 18.38 |
| DMU46 | **94.39** | 0 | **38.08** | 0.00 | **79.79** | 0.00 | **198.83** | **91.73** | **130.87** |
| DMU47 | 0.00 | **100.00** | **49.08** | 0.00 | **169.78** | **506.83** | **115.98** | **108.40** | **128.34** |
| DMU48 | 0.00 | 0 | 0.02 | 0.00 | 0.00 | 0.26 | 0.02 | 0.06 | 0.04 |
| DMU49 | **53.40** | **100.00** | 2.18 | 0.00 | 8.93 | **587.08** | 0.00 | **96.28** | **68.42** |
| DMU50 | 0.00 | **75.78** | 0.00 | 0.00 | **241.95** | **1017.14** | **530.39** | **458.64** | **548.89** |
| DMU51 | 22.06 | 0 | 0.00 | 7.36 | 0.00 | 0.00 | 5.28 | 5.35 | 0.05 |
| DMU52 | 22.19 | 0 | 0.00 | 0.00 | 17.18 | **517.39** | 10.16 | **66.73** | **44.21** |
| DMU53 | **47.19** | **100.00** | 0.00 | 0.00 | 8.09 | **699.24** | **53.55** | **41.73** | **56.68** |
| DMU54 | **77.14** | **100.00** | 0.00 | 0.00 | **73.36** | **436.96** | **47.82** | **124.08** | **109.74** |
| DMU55 | 0.00 | **86.82** | 0.00 | 0.00 | **200.97** | 7.25 | **297.05** | **325.79** | **336.10** |
| DMU56 | **93.31** | 0 | 0.00 | 0.00 | **59.60** | **131.22** | **92.77** | **106.60** | **63.51** |
| DMU57 | **76.66** | 0 | 0.00 | 0.00 | 17.18 | **517.39** | **53.55** | **106.60** | **44.21** |
| DMU58 | **47.49** | 0 | 0.00 | 0.00 | 14.56 | **1085.08** | 14.59 | **234.53** | **107.77** |
| DMU59 | 0 | 0 | 0.09 | 0.00 | 0.03 | 0.01 | 0.04 | 0.07 | 0.07 |
| DMU60 | **32.85** | **94.74** | 0.00 | 0.00 | 0.00 | **823.18** | 20.34 | **185.01** | **140.34** |
| DMU61 | **28.47** | **93.04** | **39.77** | 0.00 | 0.00 | **1700.72** | 0.03 | **122.89** | **129.15** |
| DMU62 | 0 | 0 | **88.43** | 0.00 | **108.36** | **108.35** | 108.37 | **108.40** | **158.23** |
| DMU63 | **92.43** | **100.00** | 0.00 | 0.00 | **34.48** | **440.55** | 0.00 | **126.59** | **85.12** |
| DMU64 | 0 | 0 | 0.09 | 0.00 | 0.29 | 0.00 | 0.06 | 0.06 | 0.06 |
| DMU65 | 2.76 | 0 | 0.00 | 0.00 | 74.11 | **1367.15** | 0.00 | 0.00 | 25.00 |
| DMU66 | **86.02** | 0 | **44.40** | 0.00 | 34.48 | **89.68** | **122.17** | 3.34 | 0.00 |
| DMU67 | 13.38 | 0 | 19.29 | 0.00 | 34.30 | **77.73** | **113.16** | 0.00 | 0.00 |
| DMU68 | **53.31** | **100.00** | 0.00 | 0.00 | 17.18 | **671.73** | 10.16 | **106.60** | **44.21** |
| DMU69 | **83.29** | **100.00** | **44.43** | 0.00 | 6.06 | **113.46** | 0.00 | 16.73 | 11.90 |
| DMU70 | **93.47** | 0.00 | 0.00 | 0.00 | 0.00 | **1615.83** | **36.93** | **47.95** | **77.09** |
| DMU71 | **91.64** | 0 | 0.00 | 0.00 | 0.00 | **709.99** | **55.93** | 16.88 | 9.26 |
| DMU72 | 0 | 0 | 31.49 | 0.00 | 0.00 | **727.40** | **121.78** | **69.99** | **37.18** |
| DMU73 | **83.30** | 0 | 0.00 | 0.00 | 0.00 | **972.75** | **65.23** | **50.07** | **45.23** |
| DMU74 | 0 | **100.00** | 29.21 | 0.00 | 146.10 | **65.13** | **41.71** | **41.73** | **41.73** |
| DMU75 | **74.63** | **100.00** | 0.00 | 0.00 | 0.00 | **1200.94** | 18.48 | **156.31** | **120.58** |

Supplementary Table S2: Projection Analysis of the Social Performance (%)

**Table S2 Projection Analysis of the Social Performance (%)**

|  | Input redundancy rate | | | | Output shortfall rate | | | | | | | |
| --- | --- | --- | --- | --- | --- | --- | --- | --- | --- | --- | --- | --- |
|  | S-input1 | S-input2 | S-input3 | S-input4 | S-output1 | S-output2 | S-output3 | S-output4 | S-output5 | S-outpt6 | S-output7 | S-output8 |
| DMU1 | 0.00 | 10.48 | 0.00 | 0.00 | **319.25** | 0.00 | 0.00 | **470.43** | **86.84** | 16.52 | **198.93** | **184.99** |
| DMU2 | **30.84** | **26.48** | 0.00 | 0.00 | **103.71** | 0.00 | 0.00 | 0.00 | **133.35** | 24.80 | **33.15** | **54.86** |
| DMU3 | 0.02 | 0.01 | 0.01 | 0.00 | 0.05 | 0.00 | 0.00 | 0.08 | 0.06 | 0.03 | 0.17 | 0.00 |
| DMU4 | 0.02 | 0.01 | 0.00 | 0.00 | 0.08 | 0.01 | 0.00 | 0.00 | 0.15 | 0.02 | 0.08 | 0.10 |
| DMU5 | 0.00 | 9.17 | 0.00 | 0.00 | **32.00** | 0.00 | **345.50** | **82.08** | **88.27** | 11.75 | 19.10 | 8.37 |
| DMU6 | 0.00 | 0.00 | 0.02 | 0.00 | 0.07 | 0.00 | 0.00 | 0.01 | 0.13 | 0.01 | 0.07 | 0.09 |
| DMU7 | 4.24 | 7.22 | 0.00 | 0.00 | **25.91** | 0.00 | 0.00 | 0.00 | **47.55** | 5.63 | 4.12 | 10.95 |
| DMU8 | **59.54** | 0.00 | **69.22** | 0.00 | **574.94** | 0.00 | **232.54** | **108.39** | **185.18** | **31.53** | **384.40** | **389.41** |
| DMU9 | **42.29** | **36.11** | 0.00 | 0.00 | **56.37** | 0.00 | 0.00 | **102.07** | **65.53** | 0.94 | 8.93 | 22.85 |
| DMU10 | 0.00 | 6.02 | 0.00 | 0.00 | **47.77** | 0.14 | 0.00 | 0.00 | **103.56** | 22.60 | 5.76 | 20.47 |
| DMU11 | 0.01 | 0.00 | 0.00 | 0.00 | 0.09 | 0.01 | 0.00 | 0.07 | 0.19 | 0.01 | 0.09 | 0.09 |
| DMU12 | 0 | 0.00 | 0.00 | 0.00 | 0.04 | 0.00 | 0.00 | 0.60 | 0.10 | 0.00 | 0.08 | 0.08 |
| DMU13 | 0.00 | 0.00 | 0.00 | 0.00 | **191.26** | 20.74 | **35.08** | **964.79** | **26.19** | **35.68** | **155.61** | **136.35** |
| DMU14 | 0 | **51.72** | 0.00 | 0.00 | **172.47** | 0.00 | **127.29** | **139.91** | **190.17** | **100.01** | **226.96** | **237.68** |
| DMU15 | 0 | **78.70** | 0.00 | 0.00 | 29.56 | 0.00 | 0.00 | 0.00 | **111.78** | **270.76** | **118.01** | **104.07** |
| DMU16 | **54.25** | 5.09 | 0.00 | 0.00 | 23.20 | **93.88** | 761.52 | **2000.34** | **114.05** | 0.00 | **88.32** | **66.44** |
| DMU17 | **54.78** | 0.00 | 0.00 | 0.00 | **820.57** | **34.62** | 163.43 | **172.89** | **76.17** | 24.98 | **55.98** | **67.42** |
| DMU18 | 0.00 | 24.28 | 0.00 | 0.00 | **136.88** | 0.00 | 258.84 | **381.54** | **83.41** | 3.48 | **144.71** | **146.17** |
| DMU19 | **65.41** | 23.51 | 0.00 | 0.00 | **66.44** | 0.00 | 307.62 | **112.20** | 16.96 | 0.00 | 0.00 | 3.48 |
| DMU20 | 0 | 0.00 | 0.01 | 0.00 | 0.02 | 0.00 | 0.01 | 0.01 | 0.05 | 0.01 | 0.07 | 0.07 |
| DMU21 | **70.71** | 0.00 | 0.00 | 0.00 | **94.16** | 0.00 | **72.24** | **359.37** | **94.19** | 12.13 | **166.62** | **159.61** |
| DMU22 | **72.78** | 0.00 | 0.00 | 0.00 | 21.82 | 0.00 | **222.18** | **187.19** | **187.44** | 0.00 | 19.86 | **58.24** |
| DMU23 | **42.07** | 24.64 | 0.00 | 0.00 | **247.65** | 0.00 | 27.23 | 4.97 | **134.16** | 0.24 | **146.22** | **97.97** |
| DMU24 | 0.17 | 0.00 | 0.00 | 0.01 | 0.06 | 0.00 | 0.01 | 0.40 | 0.09 | 0.00 | 0.07 | 0.29 |
| DMU25 | **37.44** | 0.00 | 0.00 | 0.00 | **715.94** | 0.00 | **89.89** | 17.52 | **272.10** | 1.11 | **45.85** | **48.39** |
| DMU26 | 0 | 0.00 | 0.00 | 0.00 | 0.12 | 0.00 | 0.00 | 0.10 | 0.07 | 0.01 | 0.13 | 0.10 |
| DMU27 | 0 | 14.34 | 0.00 | 0.00 | **1061.46** | 0.00 | 0.00 | **322.89** | **83.06** | 0.00 | **154.75** | **158.15** |
| DMU28 | 0.00 | 9.17 | 0.00 | 0.00 | **32.00** | 0.00 | **330.48** | **82.55** | **88.27** | 11.75 | 19.10 | 8.37 |
| DMU29 | 0 | **39.21** | 18.96 | 0.00 | **1235.56** | 0.00 | **70.06** | **994.05** | **142.87** | 0.00 | **142.83** | **142.83** |
| DMU30 | 0.39 | 0.00 | 0.30 | 0.00 | 0.00 | 0.00 | 0.00 | 0.25 | 0.14 | 0.14 | 0.35 | 0.60 |
| DMU31 | **31.17** | 8.36 | 0.00 | 0.00 | **98.10** | 19.26 | **715.53** | **446.60** | **42.14** | 0.00 | **102.44** | **144.34** |
| DMU32 | **100.00** | 0.00 | **65.78** | 0.00 | **166.77** | 0.00 | **74.32** | **226.88** | **64.26** | 6.20 | **90.38** | **90.38** |
| DMU33 | 0.02 | 0.01 | 0.00 | 0.00 | 0.03 | 0.00 | 2.13 | 0.25 | 0.03 | 0.00 | 0.05 | 0.05 |
| DMU34 | **74.28** | **25.76** | 0.00 | 0.00 | **43.98** | 0.00 | 0.00 | **38.43** | 16.76 | 1.06 | **89.25** | **48.77** |
| DMU35 | 0.01 | 0.00 | 0.01 | 0.00 | 0.05 | 0.00 | 0.01 | 0.24 | 0.06 | 0.00 | 0.07 | 0.06 |
| DMU36 | **100.00** | 0.00 | **41.68** | 0.00 | **403.57** | 0.00 | **117.72** | **228.49** | **403.64** | **108.34** | **183.41** | **251.18** |
| DMU37 | 0 | 0.18 | 0.00 | 0.00 | 0.07 | 0.00 | 0.00 | 0.13 | 0.00 | 0.01 | 0.11 | 0.11 |
| DMU38 | 0 | **61.29** | 0.00 | 0.00 | **111.67** | 0.00 | **189.18** | **324.48** | **80.50** | **40.81** | **82.45** | **150.29** |
| DMU39 | 0 | 0.00 | 0.04 | 0.00 | 0.03 | 0.00 | 0.02 | 0.03 | 0.07 | 0.01 | 0.07 | 0.07 |
| DMU40 | 0.00 | 0.00 | 0.02 | 0.00 | 0.05 | 0.00 | 0.00 | 0.43 | 0.09 | 0.00 | 0.08 | 0.07 |
| DMU41 | **77.99** | 0.00 | 0.00 | 0.00 | 14.34 | 0.00 | **35.75** | **386.99** | 0.00 | 0.33 | 18.66 | **49.52** |
| DMU42 | 0.09 | 0.09 | 0.00 | 0.00 | 0.02 | 0.00 | 0.00 | 1.31 | 0.06 | 0.00 | 0.12 | 0.12 |
| DMU43 | 0 | **43.86** | 0.00 | 0.00 | **282.23** | 0.00 | 13.09 | **355.52** | **76.15** | 28.49 | **84.47** | **96.92** |
| DMU44 | 0.00 | 0.00 | 0.00 | 0.00 | 0.02 | 0.00 | 0.00 | 0.01 | 0.05 | 0.01 | 0.07 | 0.07 |
| DMU45 | **53.83** | 0.00 | 0.00 | 0.00 | 29.58 | 0.00 | **535.34** | **353.75** | 0.00 | 21.34 | **56.67** | **44.73** |
| DMU46 | **63.39** | **25.59** | 0.00 | 0.00 | **128.03** | 0.00 | 0.00 | **38.36** | **201.89** | 18.03 | **138.53** | **189.67** |
| DMU47 | 0 | 0.00 | 0.00 | 0.00 | 0.56 | 0.00 | 0.15 | 1.48 | 0.33 | 0.12 | 0.17 | 0.26 |
| DMU48 | 0.00 | 0.04 | 0.02 | 0.00 | 0.00 | 0.00 | 0.29 | 0.00 | 0.02 | 0.03 | 0.05 | 0.03 |
| DMU49 | 0.00 | 0.02 | 0.00 | 0.00 | 0.14 | 0.00 | 0.66 | 0.00 | 0.00 | 0.22 | 0.58 | 0.39 |
| DMU50 | 0 | 0.00 | 24.00 | 0.00 | **206**.10 | 0.00 | **272.65** | **1050.12** | **206.14** | **109.35** | **135.79** | **192.18** |
| DMU51 | 0 | 0.00 | 0.00 | 0.00 | 0.05 | 0.00 | 0.02 | 0.03 | 0.06 | 0.00 | 0.11 | 0.09 |
| DMU52 | 0 | **57.04** | 0.00 | 0.00 | **44.98** | 0.00 | 0.00 | 0.00 | 24.80 | 13.67 | **32.98** | 28.19 |
| DMU53 | 0 | **34.48** | 0.00 | 0.00 | **72.47** | 0.00 | **140.28** | **313.37** | **90.17** | 13.34 | **63.87** | **113.77** |
| DMU54 | 0 | 0.00 | 0.01 | 0.00 | 0.03 | 0.00 | 0.01 | 0.04 | 0.05 | 0.01 | 0.07 | 0.07 |
| DMU55 | **87.98** | 0.00 | 0.00 | 0.00 | **95.36** | 0.00 | **55.68** | 0.00 | **46.19** | **354.52** | **136.16** | **113.83** |
| DMU56 | 0 | **35.17** | 0.00 | 0.00 | **44.89** | 0.00 | 29.09 | **63.77** | 29.30 | **42.86** | **58.15** | **44.93** |
| DMU57 | 0 | **39.31** | 0.00 | 0.00 | **120.75** | 0.00 | **67.29** | **114.49** | **137.08** | **53.85** | **163.70** | **120.79** |
| DMU58 | 0 | **26.98** | 22.69 | 0.00 | **112.76** | 0.00 | **196.29** | **416.33** | **112.78** | 20.00 | **344.28** | **218.72** |
| DMU59 | 0.02 | 0.00 | 0.00 | 0.00 | 0.00 | 0.00 | 0.19 | 0.12 | 0.00 | 0.00 | 0.06 | 0.05 |
| DMU60 | 0 | **58.99** | 0.00 | 0.00 | **110.63** | 0.00 | **129.78** | **191.52** | **117.45** | **48.82** | **248.85** | **254.60** |
| DMU61 | 0.00 | 0.00 | 0.01 | 0.00 | 0.02 | 0.00 | 0.02 | 0.02 | 0.05 | 0.01 | 0.07 | 0.08 |
| DMU62 | **76.56** | 0.00 | 0.00 | 0.00 | **107.32** | 0.00 | **299.13** | **772.43** | **36.42** | 3.98 | **91.76** | **142.37** |
| DMU63 | **100.00** | **78.46** | 0.00 | 0.00 | **258.68** | 0.00 | 29.49 | **110.60** | **162.21** | 0.00 | **255.40** | **258.72** |
| DMU64 | 0.39 | 0.00 | 0.00 | 0.00 | 3.04 | 0.00 | 0.00 | 0.14 | 0.08 | 0.08 | 0.00 | 0.00 |
| DMU65 | 0.00 | 0.00 | 0.00 | 0.00 | **90.85** | 0.00 | **1680.41** | **346.38** | 0.00 | **101.95** | 12.32 | **34.16** |
| DMU66 | 11.24 | 18.32 | 0.00 | 0.00 | 8.93 | 0.00 | 252.51 | **138.03** | 53.39 | 0.81 | 0.00 | 0.00 |
| DMU67 | 0.62 | **26.50** | 0.00 | 0.00 | 4.12 | 0.00 | **295.89** | 0.00 | 10.61 | 1.01 | 0.00 | 0.00 |
| DMU68 | 0 | **66.69** | 0.00 | 0.00 | **53.56** | 0.00 | 5.17 | 0.00 | 29.12 | **37.16** | **84.03** | **35.59** |
| DMU69 | 0 | 0 | 0.03 | 0.00 | 0.03 | 0.01 | 0.01 | 0.02 | 0.04 | 0.01 | 0.07 | 0.07 |
| DMU70 | 0 | 0.00 | 0.00 | 0.00 | 0.04 | 0.02 | 0.32 | 0.00 | 0.08 | 0.00 | 0.08 | 0.11 |
| DMU71 | 0 | **55.86** | 0.00 | 0.00 | 24.20 | 0.00 | **204.70** | **98.47** | **43.25** | 27.28 | 14.92 | 24.24 |
| DMU72 | **82.12** | 19.49 | 0.00 | 0.00 | 0.00 | 0.00 | **1195.05** | **235.20** | 24.53 | 0.00 | 27.37 | 7.54 |
| DMU73 | 0.65 | 0.12 | 0.00 | 0.00 | 0.00 | 0.00 | 3.50 | 0.16 | 0.25 | 0.00 | 0.25 | 0.36 |
| DMU74 | 0.00 | 5.80 | 0.00 | 0.00 | **127.15** | 0.00 | **65.44** | **238.95** | 0.00 | **34.20** | **30.45** | **35.07** |
| DMU75 | **52.67** | 0.00 | 0.00 | 0.00 | **72.07** | 0.00 | **462.11** | **530.23** | **88.92** | 4.55 | **215.44** | **203.06** |
